# Unraveling the complexity of the impact of physical exercise on male reproductive functions: a review of both sides of a coin

**DOI:** 10.3389/fphys.2024.1492771

**Published:** 2024-12-12

**Authors:** Olayinka Emmanuel Adelowo, Blessing Monica Akindele, Cecilia Adedeji Adegbola, Precious Adeoye Oyedokun, Tunmise Maryanne Akhigbe, Roland Eghoghosoa Akhigbe

**Affiliations:** ^1^ Department of Physiology, Ladoke Akintola University of Technology, Ogbomoso, Oyo, Nigeria; ^2^ Reproductive Biology and Toxicology Research Laboratory, Oasis of Grace Hospital, Osogbo, Nigeria; ^3^ Breeding and Genetic Unit, Department of Agronomy, Osun State University, Osogbo, Oyo, Nigeria

**Keywords:** exercise, male fertility, spermatogenesis, testosterone, oxidative stress, physical activity, inflammation, cytokines

## Abstract

Regular exercise is widely recognized for its numerous physical and mental benefits, but its effects on male reproductive health are less understood. This review aims to summarize the current evidence on the impact of exercise on male reproduction, including reproductive hormone regulation, spermatogenesis sperm quality, and fertility. Moderate exercise improves sperm quality, increasing count, motility, and morphology, while excessive and severe exercise may have detrimental effects. Exercise positively influences fertility by reducing oxidative stress and enhancing sperm DNA integrity. Regular physical activity regulates reproductive hormones, including testosterone, follicle-stimulating hormone, and luteinizing hormone. Exercise-induced weight management and improved insulin sensitivity also contribute to better reproductive health. In conclusion, exercise has a profound impact on male reproductive health, with moderate physical activity promoting improved hormonal balance, sperm quality, and fertility. However, severe/excessive exercise exerts negative effects. These findings imply that a balanced exercise routine, usually mild to moderate, combined with a healthy lifestyle is essential for optimal male reproductive health. However, once exercise is severe and prolonged, it could impair male reproductive health. Further research is needed to understand the mechanisms underlying the exercise-reproduction relationship fully.

## Introduction

Exercise is any physical activity that is planned, structured, and repetitive for the aim of improving or maintaining physical fitness and wellness according to Caspersen et al. (1985). Various forms of exercise involve a wide range of activities. These include aerobic exercises (e.g., running, swimming), anaerobic exercises (e.g., weightlifting, sprinting), flexibility exercises (e.g., yoga, stretching) (Dinas et al., 2011), and balance exercises (e.g., tai chi, Pilates) (Blair et al., 1989). Exercise involves the engagement of muscles and bodily systems and the regulation of intensity, duration, and frequency to achieve specific fitness goals (Haskell et al., 2007). Pate et al. (1995) explored a comprehensive framework for understanding physical activity, which includes exercise as a subset. They define physical activity as “any bodily movement produced by skeletal muscles that results in energy expenditure,” encompassing activities within and beyond structured exercise routines. Exercise encompasses a subset of physical activity that is planned, structured, and repetitive and has a final or intermediate objective to improve or maintain physical fitness. Exercise creates profound effects on metabolic pathways, hormonal balance, and immune function, contributing to the overall wellbeing of an individual (Pedersen and Saltin, 2015). Gharahdaghi et al. (2021) reported that anaerobic exercise stimulates the release of anabolic hormones such as testosterone and growth hormone, which play crucial roles in muscle growth and repair. La Vignera et al. (2012) found a positive correlation between aerobic exercise and erectile function in middle-aged men, suggesting its potential benefits for male reproductive health. However, prolonged or excessive anaerobic exercise may lead to overtraining syndrome and hormonal imbalance, thus potentially impacting adversely on male reproductive functions (Hackney, 2008).

High-intensity training increases testosterone levels, which can positively influence male reproductive functions (West and Phillips, 2012). Also, moderate exercises may improve semen quality *viz.* sperm morphology, sperm count, and sperm motility (Vaamonde et al., 2012). Regular moderate-intensity exercise has numerous benefits for male reproductive functions, including increased testosterone levels, improved sperm quality, and enhanced blood flow to the genital area (La Vignera et al., 2012). However, excessive or inappropriate exercise may cause hormonal imbalances and impairment of male reproductive processes by lowering testosterone levels, raising cortisol levels (Hackney, 2001), and inducing oxidative stress which can damage sperm DNA and impair sperm function (Aitken and Baker, 2004; 2010), potentially leading to male infertility. Prolonged periods of excessive exercise without adequate recovery may result in an overtraining syndrome characterized by fatigue, decreased performance, and hormonal disturbances that may impair male reproductive functions (Urhausen and Kindermann, 2002).

Since exercise may exert both positive and negative effects on the male reproductive function, prescription or engagement in appropriate forms of exercise remains a challenge. Thus, the present study provides in-depth information on the impact and associated mechanisms of various forms of exercises on male reproductive functions based on the available evidence from the scientific literature.

## Materials and methods

The study was based on the data obtained from a systematic search on Cochrane, Google Scholar, Pubmed, and Scopus. The Medical Subject Headings and Boolean operators used were (“exercise” OR “physical exercise” aerobic exercise” OR “anaerobic exercise” OR “dynamic exercise” OR “static exercise”) AND (“types”) AND (“male reproduction” OR “male fertility” OR “sperm” OR “semen” OR “hormone” OR “testosterone” OR “sexual function” OR “erectile function” OR “libido”) AND (“oxidative stress” OR “inflammation” OR “apoptosis” OR “mechanism”). There was no restriction to the year of publication.

### Exercise

Exercise is body movement characterized by activities like jumping, running, walking, and swimming (Winter 2009; Bartlett, 2014). This may also involve movements aided by machines or other devices like those found in wheelchair racing, cycling, rowing, kayaking, skiing, and skating (Winter 2009; Bartlett, 2014). These activities are associated with energy expenditure as much as and above 120 kJ/min (2 kW), which is about an oxygen consumption of 6 L/min in comparison with the resting rates of about 5 kJ/min (83 W) that is about an oxygen consumption of 0.25 L/min (Winter 2009). Caspersen et al. (1985) defined exercise as a planned, structured, and repetitive bodily movement.

#### Types of exercise

The American College of Sports Medicine (ACSM) defines aerobic exercise as any form of activity that engages large muscle groups that are rhythmic in pattern and maintained continuously (American College of Sports Medicine, 2013b; American College of Sports Medicine, 2013a; Wahid et al., 2016). Aerobic exercises activate muscle groups, thus utilizing energy in the form of adenosine triphosphate (ATP) (Wahid et al., 2016), and are best assessed by aerobic capacity. These forms of exercise include dancing, cycling, jogging/long-distance running, hiking, walking, and swimming (Kaminsky et al., 2013). On the other hand, anaerobic exercise is an intense physical activity of a very short duration that is fueled by the energy sources within the contracting muscles and independent of the use of inhaled oxygen as an energy source (American College of Sports Medicine, 2013b; American College of Sports Medicine, 2013a), leading to a buildup of lactic acid. Anaerobic exercises involve fast twitch muscles and include high-intensity interval training (HIIT), sprinting, and power-lifting.

#### Energy expenditure during exercise

It is important to emphasize that physical activity and energy expenditure are two different concepts. Physical activity is a behavior that leads to elevated energy expenditure beyond resting levels (Pinheiro Volp et al., 2011). Total energy expenditure (TEE) is the total amount of energy expended during 24 h, and it comprises three core components: resting energy expenditure (REE), thermic effect of food (TEF), and activity energy expenditure (AEE) (Nelms et al., 2007). The REE and the largest portion of TEE, is the obligatory energy to maintain the basic metabolic activities such as maintenance of body temperature and optimal vital organ functions (Nelms et al., 2007). REE is the energy used by a fasting person at rest in a thermo-neutral environment and it is influenced by gender, body composition, age, body temperature, energy restriction, endocrine system, and genetics (Nelms et al., 2007).

#### Benefit of exercise

Exercise is a commonly prescribed therapy in health and in diseased states. There is irrefutable evidence demonstrating the positive effects of exercise in the prevention and management of several pathologies (Macera et al., 2003). Exercise comprises a series of sustained muscle contractions, of either long or short periods, depending on the nature of the physical activity (Boström et al., 2013). Muscle-strengthening activities increase/maintain muscle mass and strength (Boström et al., 2013). Strong muscles and ligaments reduce the risk of joint and low back pains by keeping joints in proper alignment (Boström et al., 2013). Also, increased levels of physical activity and fitness reduce mortality by about 20%–35% (Blair et al., 1989; Macera et al., 2003), reduce morbidity, and improve fertility profile (Bouchard et al., 1994; Warburton et al., 2006). In addition, exercise prevents pulmonary and cardiovascular diseases such as chronic obstructive pulmonary disease, hypertension, metabolic disorders (type II diabetes, dyslipidemia, and obesity), chronic fatigue syndrome, osteoporosis, rheumatoid arthritis, cancer, depression (Pedersen and Saltin, 2015), and age-related mental decline (Laurin et al., 2001) and improves the quality of sleep, learning and memory (van Praag et al., 1999), cognitive function (Dishman et al., 2006), and functional recovery from brain injury (Grealy et al., 1999). Exercise also induces neurogenesis in the adult dentate gyrus (van Praag et al., 1999) and can contribute to remodeling hippocampal synaptic circuits and enhancing cognitive function.

#### Adverse effects of exercise

Prolonged exercise may impair the hypothalamic-pituitary-testicular axis (Hackney, 2001). This hypothesis is supported by data that revealed a reduced maximum rise in the levels of pituitary hormones (corticotrophin and growth hormone), cortisol, and insulin after an exhaustive exercise (Urhausen et al., 1995), and a negative association between testosterone levels and training volume in men participating in chronic endurance training (MacKelvie et al., 2000). This alteration may result in a reduction in circulating testosterone levels (Hackney, 2001); however, limited information is available due to a lack of relevant human studies. Nonetheless, mild physical exercise is non-pharmacological management for sleep disorders (Sleep-enhancing effect of exercise), although excessive exercise and overtraining are associated with insufficient or poor sleep (Santos et al., 2007). Sleep disorders may occur due to a distortion in circadian rhythms, psychobehavioral (mood, behavior, and cognitive) alteration accompanying overtraining, and an imbalance in the neuroendocrine signaling (Smith, 2000). Excessive exercise may cause elevated circulating concentrations of sympathetic-dependent catecholamines and increased cortisol secretion by the adrenal cortex (Smith, 2000), therefore impairing the hypothalamic-pituitary-testicular axis and testosterone release.

### Exercise and male fertility

Optimal physical activity and exercise promote general wellbeing. Exercise is a useful strategy employed to promote physical and psychological wellbeing, prevent chronic diseases, promote weight loss, and enhance sleep quality (Milani et al., 2011). Exercise is beneficial to male reproductive health regardless of age, sex, or physical ability. In addition, physically active individuals have better semen quality and hormone levels than sedentary subjects (Vaamonde et al., 2012).

#### Exercise and testicular steroidogenesis

**FIGURE 1:**
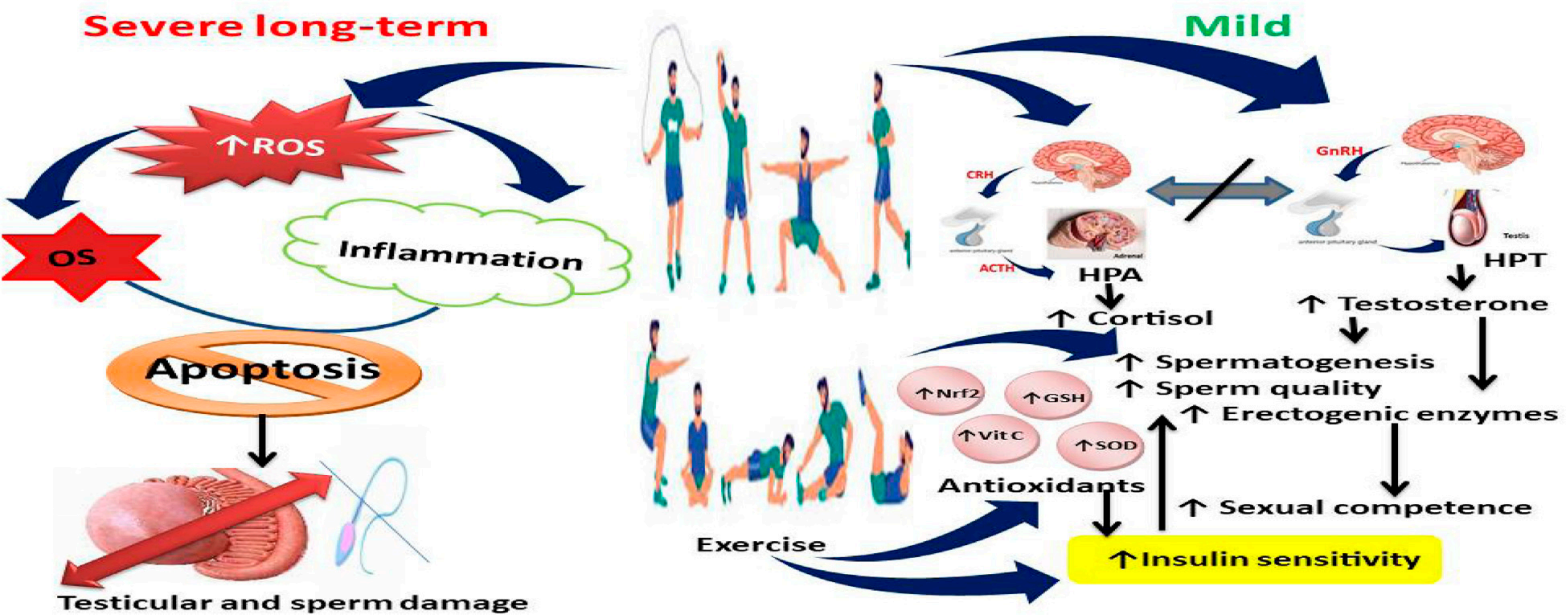
Effect of exercise on testosterone secretion and spermatogenesis. Although severe exercises upregulate reactive oxygen species (ROS) generation, leading to oxidative stress (OS) and inflammation that may culminate in apoptosis and testicular and sperm cell damage, mild exercises dissociate the nexus between hypothalamic-pituitary-adrenal (HPA) axis and hypothalamic-pituitary-testicular (HPT) axis, leading to increased cortisol and testosterone, enhanced antioxidant levels and activities and insulin sensitivity, and increased spermatogenesis, sperm quality, and male sexual competence. Vit C, Vitamin C; SOD, superoxide dismutase; GSH, reduced glutathione; Nrf2, nuclear factor erythroid 2-related factor 2.

Exercise prevents age-related disorders, such as diabetes, hypertension, and hyperlipidemia (Kushkestani et al., 2022). Sex steroid hormones decrease with aging (Ketchem et al., 2023), thus increasing the risk of incident metabolic syndrome (Jia et al., 2023). Studies have shown that acute and chronic exercises alter circulating sex steroid hormone levels (Dote-Montero et al., 2021). Dehydroepiandrosterone (DHEA) is a precursor of sex steroid hormones and is converted by 17β-hydroxysteroid dehydrogenase (HSD) and 3β-HSD enzymes to testosterone (Ajayi and Akhigbe, 2020a; Oyedokun et al., 2023; Besong et al., 2024; Besong et al., 2023a). However, sex steroid hormones are produced primarily by the gonads to act on target organs/tissues like the liver, heart, kidney, bones, and brain. Brownlee et al. (2005) revealed that exercise improved circulating testosterone levels by inhibiting the negative correlation that usually exists between cortisol and testosterone, resulting in a positive relationship between cortisol and testosterone due to adrenal cortex contribution to testosterone secretion and/or dissociation of testosterone from sex hormone-binding globulin (Figure 1). Liu et al. (2009) reported that although overexertion may suppress testosterone secretion, mild physical activity enhances testosterone secretion by improving insulin sensitivity and upregulating the activities of steroidogenic enzymes. The rise in circulating testosterone after an acute resistance exercise bout has also been attributed to the upregulation of androgen receptors (Hooper et al., 2017).

While there seems to be a controversy on the effects of exercise on fertility in males, there is enough evidence to conclude that exercise has considerable effects on testicular steroidogenesis. The findings of Yi et al. (2020) showed that moderate-load exercise slightly mitigated obesity-induced decrease in testicular testosterone synthesis in Wistar rats. This occurred to the downregulation and decreased expression of steroidogenic enzymes steroidogenic factor-1; StAR: steroidogenic acute regulatory protein; and P450scc: P450 side chain cleavage (Cyp11a1) (Yi et al., 2020). Similarly, mild exercise was shown to induce reversible positive changes in the testosterone/17β-estradiol (T/E_2_) ratio when combined with food intervention in Wistar rats (Santillo et al., 2020). However, Manna et al. (2003) reported that chronic intense exercise led to the diminution of LH, FSH, and testosterone also in Wistar rats. The decrease in testicular testosterone was associated with a decrease in the activity of 3*β*-hydroxy-steroid dehydrogenase (3*β*-HSD), 17β-hydroxysteroid dehydrogenase (17*β*-HSD) (Manna et al., 2003). Though, there were significant changes in Western blot analysis of cytochrome P450scc (*Cyp11a1*) gene expression, intensive swimming exercise induced an observable decrease in the expression of testicular StAR (Jana et al., 2014).

From the foregoing, it is evident that more studies are reporting the adverse effects of exercise on testicular steroidogenesis and male sex hormones. The mechanism of this effect has been explored. The decrease in testicular steroidogenic markers following exposure to chronic intense exercise is associated with a rise in the levels of MDA, and conjugated dienes with a decrease in GSH, CAT, SOD, GPx, and GST; and caspase-3 dependent apoptosis (Manna et al., 2003). Similar patterns of findings were reported by Jana et al. (2014), Santillo et al. (2020), and Yi et al. (2020). The metabolic implication of this effect and mechanism has also been reported. The observed compromise of the antioxidant defense system and increased caspase-3 activities reduction was related to the reduction in glucose-6-phosphate dehydrogenase and depletion of the mitochondrial membrane potential and intracellular ATP generation (Jana et al., 2014). The vasodilatory effect of testosterone (Akorede et al., 2024) also promotes the clearance of toxic metabolite and maintains optimal redox status in the testis, thus further enhancing androgen production and spermatogenesis.

#### Exercise and hypothalamic-pituitary-adrenal axis

The hypothalamic-pituitary-adrenal (HPA) axis and autonomic nervous system participate in the maintenance of homeostasis. In response to the stress of rigorous exercise, the HPA axis reacts by elevating plasma cortisol and catecholamine levels (Leistner and Menke, 2020). In addition, reports have shown that sustained physical conditioning in an athlete may be associated with activated or suppressed HPA axis in response to exercise (Anderson et al., 2019). Conversely, well-trained athletes exhibit a chronic yet mild form of hypercortisolism at baseline that may be an adaptation to constant exercise (De Luccia, 2016). The proinflammatory cytokine, IL-6, is activated during exercise; additionally other proinflammatory factors such as myeloperoxidase, PMN elastase, and IL-10 increase in response to intense exercise (Suzuki et al., 2020). More so, testicular microtrauma and temperature rise may cause a decline in testosterone biosynthesis (Akhigbe et al., 2022a).

#### Exercise, spermatogenesis, and sperm quality

Spermatogenesis, a complex metabolic process that ensures the production of quality male gametes (Oyedokun et al., 2023), and sperm motility, sperm morphology, and sperm count are important indices for assessing male infertility (Ajayi and Akhigbe, 2020c). As observed in people with sedentary lifestyles and obesity (Salas-Huetos et al., 2021), physical inactivity is associated with reduced semen quality (Sun et al., 2019). There is accumulating evidence that a sedentary lifestyle can adversely affect spermatogenesis (Sharpe, 2010). However, data on the effects of exercise on testosterone and spermatogenesis are diverse. Studies have shown that long-term endurance exercise training can decrease the production of testosterone (Hackney and Lane, 2018), which in turn could impair spermatogenesis (Ajayi and Akhigbe, 2020b). However, mild exercise may be beneficial. Exercise improved sperm motility and morphology as well as reduced sperm DNA damage in obese rats fed on a high-fat diet by attenuating ROS generation and reducing mitochondrial membrane potential via the upregulation of ghrelin and stem cell factors (Alhashem et al., 2014), modulation of mir-34a/SIRT1/p53 signaling (Heydari et al., 2021), and downregulation of proliferative cells, ZO-1, occludin, and gap junction protein Cx43 (Elmas et al., 2022). Exercise has also been shown to improve insulin sensitivity and promote the utilization of cholesterol (Muscella et al., 2020; Iaccarino et al., 2021), thus enhancing testosterone production, which in turn facilitates spermatogenesis (Akhigbe et al., 2023).

In various human studies on moderate aerobic exercise, generally, positive effects have been noted on sperm parameters. Vaamonde et al. (2012) reported high sperm motility in males with regular moderate physical activity compared to their sedentary counterparts. Thus, the improvement is related to improved blood flow and oxygenation in testes organs that support optimal sperm development. On the contrary, sperm count and motility can be reduced in men with long and vigorous endurance exercises. This has been related to, sometimes discussed, exercise-induced male infertility, basically as a result of increased scrotal temperature and augmented oxidative stress in tenuous physical activity.

Resistance training itself is known to raise the levels of testosterone, which forms the critical material and regulatory element in the process of spermatogenesis (Ajayi and Akhigbe, 2020b). In the study carried out by Hooper et al. (2017), resistance exercise in combination with adequate nutrition improved androgenic responses, with possible enhancement of spermatogenesis and sperm quality. Likewise, resistance exercises in animal studies have shown the potential to increase testicular activity and improve sperm parameters in rodents. Hayes et al. (2015) showed improved sperm motility and morphology in rats that underwent resistance exercise intervention. Similar benefits from combined exercise interventions were also found in animal models. According to Wan et al. (2024), voluntary wheel running, an exercise combining the two aforementioned types of physical activity, that is, aerobic and resistance exercises, improved antioxidant capacity in rodents, which might also improve sperm quality. Exercise also reprograms male reproduction by regulating adiposity and gonadal fat, and attenuating oxidative stress, thus improving sperm quality and male fertility (Santos et al., 2015).

**FIGURE 2:**
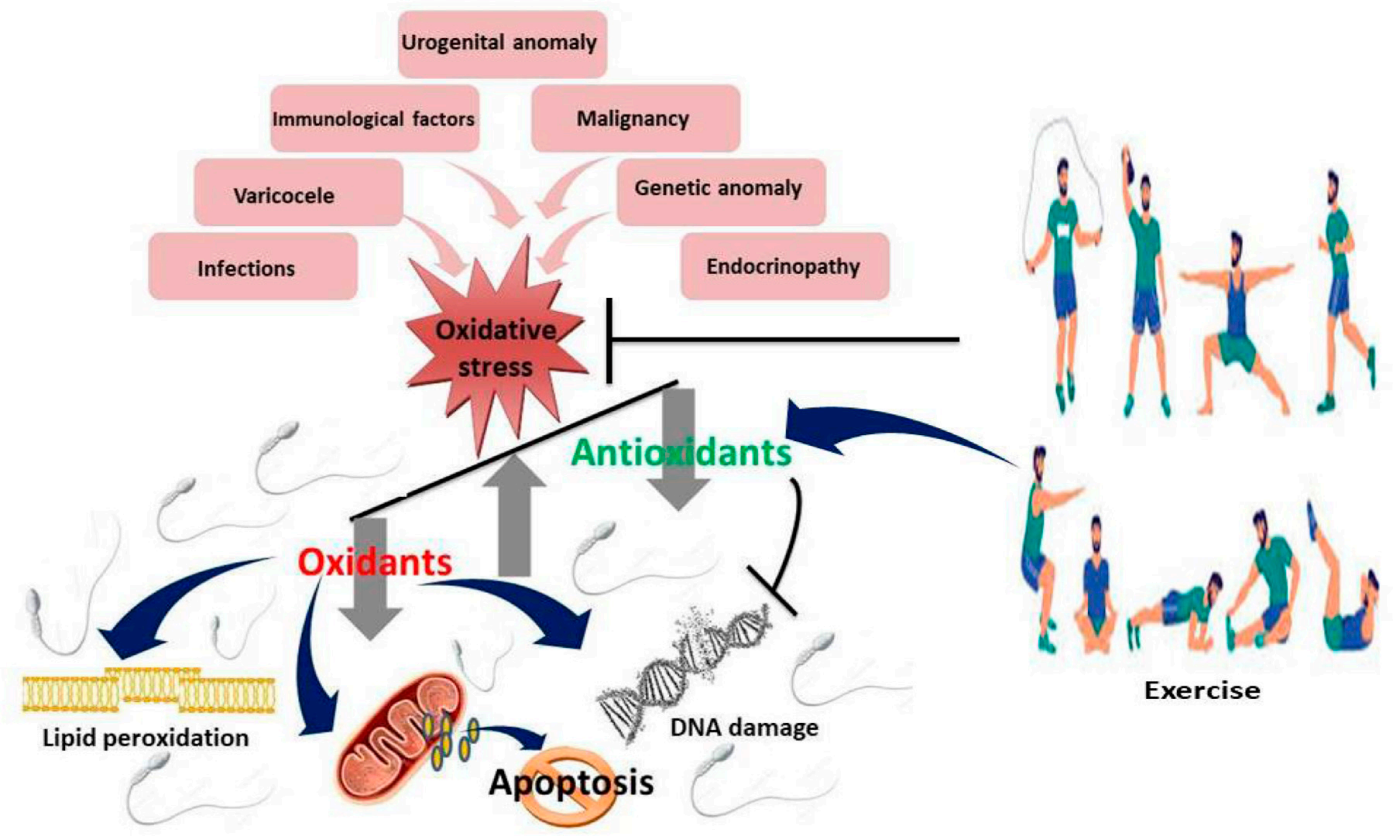
Effect of exercise on sperm quality Mild to moderate exercises inhibits oxidative stress by upregulating antioxidants and suppressing oxidants, thus downregulating lipid peroxidation, DNA damage and apoptosis.

Exercise plays its role in spermatogenesis (Figure 1) and sperm quality (Figure 2) through complex physiological ways. Both testosterone, follicle-stimulating hormone, and luteinizing hormone, which are essential for the production and maturation of sperm, are regulated according to the level of exercise. Testicular blood flow is increased with exercise, and this is important for delivering nutrients and removing waste products of spermatogenesis. Moreover, adaptations driven by exercise at various levels, the induction of antioxidant defenses, and the repression of oxidative stress play a critical role in maintaining both the quality and sperm count (Elmas et al., 2022; Akhigbe et al., 2023).

#### Exercise and male sexual competence

Erectile dysfunction (ED), a persistent inability to achieve and/or sustain an optimal erection for satisfactory sexual performance, may be triggered by impaired blood flow and pressure on the penile nerves as a result of extended periods on the saddle. Sexual health and competence are integral dimensions of males’ wellbeing (Ajayi and Akhigbe, 2020d; Besong et al., 2024; Besong et al., 2023b). Exercise has traditionally been claimed to be useful not only for general health but also for enhancing sexual functioning. This essay elucidates evidence-based information on how various kinds of exercise affect male sexual behavior and competence. Exercise modulates the levels of many hormones, such as testosterone, cortisol, and endorphins, which are involved in sexual behavior and competence. Through regular exercise at moderate intensities, increased testosterone levels and decreased cortisol levels occur, reflecting favorably on libido and sexual performance (Hayes et al., 2015).

Pudendal nerve neuropraxia causes numbness and an impaired sensation during ejaculation (Aoun et al., 2021). Also, ED is associated with lowered NO release and/or its bioavailability to the corporeal smooth muscle (Adeyemi et al., 2022). More so, ED is promoted by a surge in superoxide anions generation and contractile factors (Kaltsas et al., 2024). Human studies revealed that exercise improves endothelial function by up-regulating eNOS protein expression and phosphorylation (Gilligan et al., 1994; Green et al., 2004). Although improvement in NO vasodilator function has not been well-reported in healthy individuals, a higher level of training may lead to improvement. Short-term training promotes NO bioactivity that in turn regulates the shear stress associated with exercise (Green et al., 2004). While the increase in NO bioactivity dissipates within weeks of training stoppage, if exercise is continued, the short-term functional adaptation is succeeded by NO-dependent structural changes, leading to arterial remodeling and structural normalization of shear (Green et al., 2004), therefore promoting penile endothelial function and erection.

Repeated bouts of exercise over weeks or months upregulate endothelial NO bioactivity. Experimental studies reported improved endothelium-dependent vasodilation within a week of exercise (Maiorana et al., 2003). Although vasodilator function continues to improve as exercise persists for several weeks, it may decline with long-term training, possibly due to structural adaptation that is partly endothelium-dependent (Maiorana et al., 2003). Interestingly, individuals with initially impaired endothelial function tend to be more responsive to exercise than healthy individuals (Gilligan et al., 1994; Maiorana et al., 2003). The increased circulating NO is associated with enhanced antioxidant effects, thus promoting penile endothelial function and erection.

Cardio exercises such as running, swimming, and cycling have been implicated in the restoration of sexual function in men. These exercises enhance blood flow and vascular function throughout the body, including the genital area, and subsequently, cardiovascular health. Improved blood flow to the penis can lead to better erectile function and overall sexual performance (Riachy et al., 2020). Another exercise method significantly linked to the sexual health of men is resistance training, such as weightlifting. It has been suggested that resistance training elevates testosterone levels; important for libido and erectile function. In addition, resistance training enhances muscle strength and endurance apart from an increase in overall stamina and physique for better sexual performance (Hildreth et al., 2018).

Yogas, tai chi, and other forms of mind-body exercises follow the principles of relaxation, reducing stress, and the induction of mindfulness. These activities are often linked to superior sexual function since reduced levels of stress and anxiety, well-established inhibitors of sexual performance, are reduced (Dhikav et al., 2010). Mind-body exercise offers greater body awareness and control, which results in increased sexual pleasure and gratification (Dhikav et al., 2010). Pelvic floor exercises, in particular the Kegel’s exercise, strengthen the muscles that support penile erectile function; hence, they prevent erectile dysfunction and ensure better control over ejaculation. Exercise is most useful in men suffering from sexual dysfunction after prostate surgery or due to age-related changes (Hildreth et al., 2018).

In addition to physiological effects, it has considerable psychological benefits that help in supporting sexual health. The enhanced self-esteem and reduced anxiety coupled with the improved mood that comes as a result of regular exercise may contribute to increased sexual desire and satisfaction (Herbert et al., 2020). Altogether, various forms of exercise have varying benefits on sexual behavior and competence among males. Cardiovascular exercises enhance blood flow and erection; resistance exercises promote testosterone levels and raise physical performance; mind-body exercises reduce stress and anxiety; pelvic floor exercises may strengthen erectile muscles; and, in sum, exercise exerts multiple different positive effects on hormones and psychological factors involved in sexual health. Administered regularly, exercise might contribute not only to physical but also to psychosexual wellbeing in men.

The impact of exercise on vascular tone transcends vasodilation and enhancement of testosterone production. The upregulated testosterone improves endothelial function, energy levels, muscle strength, and the release of stimulatory neurotransmitters like dopamine, nitric oxide, and oxytocin that in turn enhance libido and male sexual competence (Akhigbe et al., 2022b; Adeyemi et al., 2024).

**FIGURE 3:**
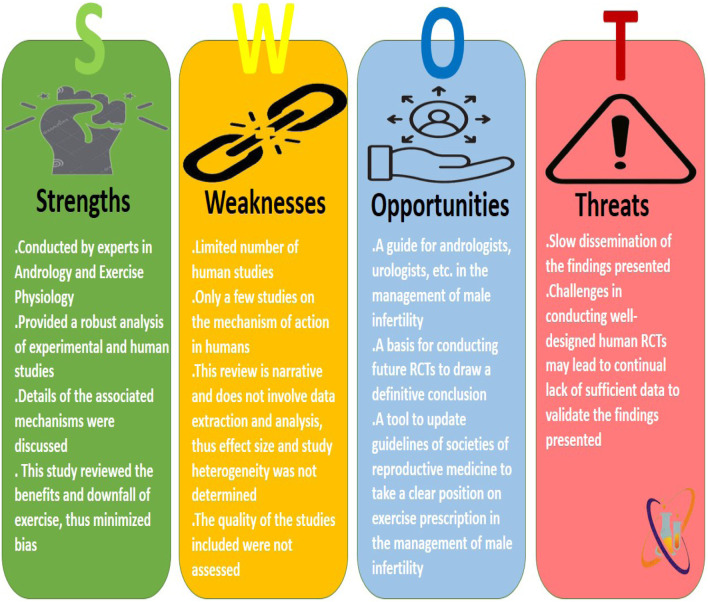
The Strengths, Weaknesses, Opportunities, and Threats (SWOT) analysis of the present study.

Despite the robustness of this study, there are some limitations. First, there is a paucity of human randomized controlled trials evaluating the impact of graded exercises on male reproductive function, hence limiting the depth of our report. Also, most studies that explored the associated mechanisms of exercise on male fertility were on animal rodents, which limits the extrapolation of these findings directly to humans. Nonetheless, the present study provided detailed information based on experimental and human studies, explaining the benefits and downfalls of varying grades of exercise. Details of our SWOT analysis are presented in Figure 3.

## Conclusion and future perspectives

Data available in the literature provide pieces of evidence that reveal that exercise exerts a dual effect on male reproductive function. Severe long-term exercise impairs testicular steroidogenesis by enhancing ROS generation, while mild short-term exercise improves testicular steroidogenesis, spermatogenesis, and sexual competence by increasing insulin sensitivity, downregulating ROS generation, and modulating cortisol-testosterone crosstalk. Nonetheless, clinical studies validating these findings and exploring other mechanisms associated with the benefits of mild exercise are recommended.
